# Rosaceae Fruit Development, Ripening and Post-harvest: An Epigenetic Perspective

**DOI:** 10.3389/fpls.2017.01247

**Published:** 2017-07-17

**Authors:** Silvia Farinati, Angela Rasori, Serena Varotto, Claudio Bonghi

**Affiliations:** ^1^Department of Agronomy, Food, Natural Resources, Animals and Environment, University of Padova Agripolis Legnaro, Italy; ^2^Centro Interdipartimentale per la Ricerca in Viticoltura e Enologia, University of Padova Conegliano, Italy

**Keywords:** epigenetics. DNA methylation, histone modifications, transcriptional regulation, fruit patterning, transcription factors

## Abstract

Rosaceae is a family with an extraordinary spectrum of fruit types, including fleshy peach, apple, and strawberry that provide unique contributions to a healthy diet for consumers, and represent an excellent model for studying fruit patterning and development. In recent years, many efforts have been made to unravel regulatory mechanism underlying the hormonal, transcriptomic, proteomic and metabolomic changes occurring during Rosaceae fruit development. More recently, several studies on fleshy (tomato) and dry (Arabidopsis) fruit model have contributed to a better understanding of epigenetic mechanisms underlying important heritable crop traits, such as ripening and stress response. In this context and summing up the results obtained so far, this review aims to collect the available information on epigenetic mechanisms that may provide an additional level in gene transcription regulation, thus influencing and driving the entire Rosaceae fruit developmental process. The whole body of information suggests that Rosaceae fruit could become also a model for studying the epigenetic basis of economically important phenotypes, allowing for their more efficient exploitation in plant breeding.

## Introduction

The biological and economic importance of the Rosaceae family has been well described in several reviews, summing up the phylogenetic, botanical, physiological and genomic features of its different species. More than 2500 species from 90 genera belong to the Rosaceae family and many are economically important crops producing edible fleshy fruits (e.g., apple, apricot, cherry, peach, pear, plum, raspberry, and strawberry), nuts (e.g., almond), and ornamentals (e.g., rose) (Yamamoto and Terakami, 2016). However, the importance of edible Rosaceae crops is mainly due to the economic significance of their fruits that provide unique contributions to a healthy diet for consumers. In this context, their role as sources of phytochemicals and antioxidants has been well testified for several decades (Swanson, 1998; Shulaev et al., 2008). For these reasons the numerous efforts to collect as many as resources as possible for the Rosaceae research community, including large amounts of genomic, genetic and breeding data are justified. In recent years it has been observed an increase of the application of molecular technologies in Rosaceae research: genetic and bioinformatic tools, including annotated whole genome sequences, transcriptomes, proteomic and metabolomic information, have become essential in basic and applied research, also focusing attention on the dynamics driving fruit development and ripening (**Table 1**) (Jung and Main, 2014). For example, as reported in three model species of Rosaceae, apple, peach, and strawberry, characteristic genetic linkage maps and several markers associated to traits of economic importance, such as fruit quality and resistance to pests and diseases, have been well described in numerous works (Shulaev et al., 2008; Cao et al., 2016; Xiang et al., 2016). However, although a large amount of information has been produced to define some aspects of Rosaceae fruits, little has been clarified on the molecular mechanisms that drive gene regulation during their development, from the early stages till harvesting. Moreover, since post-harvest events deeply affect the product quality and consequently the commercial value of fruits, a lot of reports in the last few years have highlighted the various detrimental effects of stresses on post-harvest product quality, with special emphasis on its practical management, and the possibility of using ‘omics’ tools.

**Table 1:** Summing of main botanical, physiological and genomic properties of four Rosaceae fruits considered model species in Rosaceae research.

	Fruit type	Physiology	Genome size^a^	Ploidy^a^	RNAseq data^b^	Endo-reduplication	Genetic transformation
Peach (*Prunus persica*)	Drupe	Climacteric	265 Mb	Diploid	Yes	Yes	Yes^∗^
Apple (*Malus × domestica*)	Pome	Climacteric	750 Mb	Diploid or triploid	Yes	No	Yes^∗∗^
Woodland strawberry (*Fragaria vesca*)	Achene	Non-climacteric	240 Mb	Diploid	Yes	Yes	Yes^∗∗∗^
Black raspberry (*Rubus occidentalis*)	Drupeole	Non-climacteric	240 Mb	Diploid	_	_	Yes^∗∗∗∗^

*The specific information are obtained querying the following www.rosaceae.org^*a*^ and www.ncbi.nlm.nih.gov^*b*^ databases. The underscore indicates that information are not available. ^∗^Pérez-Clemente et al. (2004), ^∗∗^Malnoy et al. (2010), ^∗∗∗^Husaini and Srivastava (2006), and ^∗∗∗∗^Swartz and Stover (1996).*

The availability of molecular resources in different Rosaceae species has increased the genetic data collection from fruit set to pre-harvest ripening, resulting as being an important research tool (Shulaev et al., 2008). However, these notions on fleshy fruit systems belonging to this family are again limited, if compared to those obtained from molecular and physiological investigations on model tomato. In this context and summing up the results obtained so far, this review aims to collect the available information on mechanisms, overall epigenetic mechanisms, that may provide an additional level in gene transcription regulation, thus influencing and driving the entire fruit developmental process. The main epigenetic mechanisms involved in fruit growth have been recently reviewed by several authors (Ecker, 2013; Osorio et al., 2013; Teyssier et al., 2015; Gallusci et al., 2016). Because these papers have mainly focused their topic on tomato fruit, this review aims to focus our attention on fruits belonging to Rosaceae family, describing in a single paper the major players involved in these developmental dynamics from fruit set to post-harvest, by comparing the available data with that obtained in fleshy or dry fruit models (e.g., tomato and Arabidopsis siliques).

## Epigenetic Control In Plant/Fruit Development

In Rosaceae fruit crops, like in other plant families, several studies have shown that both fruit set and development (including ripening) rely on the establishment and maintenance of differential gene expression patterns (Janssen et al., 2008; Bonghi et al., 2011; González et al., 2016) determined by the combined effects of complex hormonal (endogenous) and environmental (exogenous) mediated controls on transcription, operating at different levels during fruit developmental processes (Klee and Giovannoni, 2011). Although the basic principles of gene transcriptional regulation are universal among eukaryotic organisms, it is well known that the DNA packaging into the chromatin determines a more sophisticated and specific regulatory option, essential for eukaryotic organisms to express and modulate genes in diverse patterns required for a higher biological complexity (Lauria and Rossi, 2011). Mechanisms leading to the alteration of chromatin structure and the consequent stable changes in gene expression, without modifications of the underlying DNA sequence, are defined as epigenetic mechanisms (Bonasio et al., 2010). They include DNA methylation and histone Post-Translational Modifications (PTMs) processes, exhaustively described in recent reviews (Pikaard and Mittelsten Scheid, 2014; Niederhuth and Schmitz, 2017). Both mechanisms are responsible of a specific cell/tissue epigenome establishment, transmittable by DNA replication and determining of peculiar gene expression patterns and phenotypes (Wang and Köhler, 2017). Studies in the model Arabidopsis and fruit crops, including for the latter tomato as model for fleshy fruit, have confirmed the relevance of epigenetic mechanisms in the control of plant ontogenesis (Hsieh and Fischer, 2005; Lauria and Rossi, 2011; Gallusci et al., 2016). Moreover, an epigenetic influence in controlling of traits with a high agronomic interest, such as flowering time, heterosis and fruit ripening process, was also testified (He et al., 2011; Zhong et al., 2013; Liu et al., 2015).

### DNA Methylation Pathways

Differently from animals, DNA methylation in plants can occur at cytosine both in symmetrical (CG or CHG) and non-symmetrical (CHH) contexts (with H: A, T or C) and is driven by three main classes of DNA methyltransferases. The DNA methyltransferases 1 (MET1), which is an ortholog of the mammalian Dnmt1, is responsible for maintenance of CG methylation in cooperation with the VARIANT IN METHYLATION (VIM) proteins. CHG methylation is mainly maintained by a self-reinforcing loop between the plant-specific CHG DNA MTase CHROMOMETHYLASE 3 (CMT3) and the H3K9 MTase KRYPTONITE (KYP), also known as SUPPRESSOR OF VARIEGATION 3–9 HOMOLOG 4 (SUVH4) and its close homologs SUVH5 and SUVH6 (Lindroth et al., 2001; Du et al., 2012, 2014, 2015). The establishment of DNA methylation in all sequence contexts and the maintenance of CHH methylation in plants utilizes a plant-specific RNA directed DNA methylation (RdDM) pathway to guide the DOMAINS REARRANGED METHYLTRANSFERASE 2 (DRM2). Current knowledge about RdDM derives essentially from researches conducted on *Arabidopsis thaliana*. This pathway involves the biogenesis of siRNAs (short interfering RNAs), which requires Pol IV, RDR2 and DCL3, and *de novo* methylation, which requires plant Pol V-dependent scaffold RNAs, AGO4-bound 24-nt siRNAs, and the *de novo* DNA methyltransferase DRM. Many other dedicated proteins participate in RdDM, whose precise roles have not so far been fully clarified (Matzke et al., 2015). More recently several “non-canonical RdDM” mechanisms that function to initiate gene silencing and modify chromatin were described (reviewed in Cuerda-Gil and Slotkin, 2016). An essential chromatin factor for plant DNA methylation is the Snf2 family nucleosome remodeler DDM1 (Jeddeloh et al., 1999). DDM1 promotes methylation in all sequence contexts independently from RdDM and mediated RdDM-independent CHH methylation, which is catalyzed by the chromomethylase CMT2. In Arabidopsis, the lack of CMT2, a homolog of CMT3, caused a consistent loss of CHH methylation and experimental evidences indicated that CMT2 is responsible for the DDM1-mediated, sRNA-independent CHH methylation (Zemach et al., 2013). CMT2 has been shown to *de novo* methylate both CHH and CHG sites *in vitro*, in contrast to CMT3 that is responsible for maintaining CHG site methylation (Stroud et al., 2014).

Genome-wide studies have shown that a specific DNA methylation distribution in three contexts depends upon TE sequences occupancy in Arabidopsis and in other relatively small plant genomes (Kim and Zilberman, 2014). In these genomes a higher density of TE sequences and DNA methylation in pericentromic regions is commonly present while this is not the case for much larger plant genomes, which inexplicably show a high density of TE sequences and DNA methylation throughout the chromosome arms. However, in both small and large plant genomes, DNA methylation over TEs is known to spread to nearby genes, thus affecting their expression (Hollister and Gaut, 2009). Together with TEs, genes contribute with gene body methylation to shape genomic DNA distribution. Genes with moderate expression levels show CG sites methylation (as well as CHG sites in conifers) over their coding sequences. Although its role has not been clearly assessed, gene body methylation has been proposed to have different homeostatic functions, such as silencing of cryptic promoters, effect on splicing efficiency, reduction of gene expression variability (Takuno and Gaut, 2013; Bewick and Schmitz, 2017; Zilberman, 2017). To erase DNA methylation, plants use DNA glycosylases that excise methylated cytosines: Arabidopsis has four glycosylases, ROS1, DML2, and DML3, which are expressed throughout development and are generally functionally redundant, with their main function likely being to maintain genic regions containing TE or other repeat sequences free of DNA methylation. DME is a plant glycosylases, expressed exclusively in the central cell of the embryo sac and the pollen vegetative cell, essential for the extensive maternal and more limited paternal-specific DNA demethylation of TEs that underlies genomic imprinting phenomenon in the endosperm tissue (Calarco et al., 2012; Ibarra et al., 2012).

Many reports reviewed mechanisms of plant DNA methylation and how they are interconnected with other epigenetic pathways, like the small RNAs responsible for guiding DRMs in CHH methylation in the RdDM process (see below and Saze et al., 2012), or the requirement for H3K9 methyltransferases KRYPTONITE (KYP/SUVH4), SUVH5 and SUVH6, as shown in Arabidopsis, in CHG methylation (Johnson et al., 2007; Du et al., 2012; Kawashima and Berger, 2014; Bewick and Schmitz, 2017). Several genome-wide analyses have revealed that DNA methylation distribution in plant genomes is mainly associated to tandem and dispersed repeats (Chen et al., 2010) and that DNA methylation regulates the gene modulation of loci expressed in a tissue-specific manner throughout the plant development cycle. Although this epigenetic mark is considered the most stable among epigenetic marks, many works demonstrated its extreme dynamicity in plant response to environment (Sahu et al., 2013). In opposition to model Arabidopsis, in which the 5MeC genome distribution and the role of the players involved in 5MeC establishment is well documented, in fruit crops few of these notions are clarified and few examples are reported. Furthermore in these crops the role of 5MeC epigenetic mark was also exploited for different purposes: for example in grapevine a different methylation profile, determined by MSAP, was employed to distinguish between different grape clones (Ocaña et al., 2013), in sweet orange an expression analysis of genes involved in DNA methylation and a global DNA methylation distribution was detected (Xu et al., 2015) and in banana a putative correlation between DNA methylation level and genome size was determined (Msogoya et al., 2011). These observations are added to the numerous data obtained in the last decades relative to tomato, revealing a direct link between fruit size, ripening and an epigenetic control (Manning et al., 2006; Liu D. D. et al., 2012) and an unusual 5MeC distribution if compared to other species (Zhong et al., 2013).

### Chromatin Dynamics

Post-Translational Modifications of histones affect chromatin condensation and structure and consequently have a role in the epigenetic control of gene regulation. The impact of chromatin organization on the transcription process is mainly due to the degree of packaging of genomic DNA around the nucleosome units. Alteration of nucleosomes organization, in terms of stability and positioning, can influence the DNA accessibility to regulatory proteins or protein complexes (Kouzarides, 2007). PTMs of histones consist of chemical modifications (acetylation, methylation, phosphorylation, and ubiquitination) and many other covalent modifications of various amino acids supported by a plethora of enzyme complexes (Kouzarides, 2007). In Eukaryotes, nucleosome histones are chemically modified at many residues and the histone code hypothesis proposes that combinatorial marks could specify different regulatory outcomes. In plants, depending on the type and combination of PTMs in canonical histone tails, it is possible to define a specific chromatin state associated to an active or repressive gene modulation: for example histone acetylation appears to be associated positively with gene expression, whereas histone methylation may be the cause of either gene repression or activation depending on the residue modified (Sequeira-Mendes et al., 2014). In detail, deacetylation of histone H3 together with di-methylation of Lys 9 (H3K9me2) or tri-methylation of Lys 27 (H3K27me3) are associated with a repressive chromatin conformation and hence with repressed repetitive sequences or genes, respectively, while acetylated histones along with H3K4me3 and H3K36me3 are examples of an active chromatin state and therefore correlated with actively transcribed genes (Kim et al., 2007). Histone readers, proteins that can bind modified histones through their chromatin-binding domains (e.g., chromo-, bromo-domains) can form higher order complexes with other effectors, such as histone modifiers (e.g., acetyl- and methyltransferases), to translate a histone mark into a transcriptional output. Among the several specialized enzymes involved in histone mark dynamicity it is possible to collocate the Histone Methyl Transferase (HMT) and Polycomb Group (PcG) proteins for the deposition of methyl group addition and Histone Acetyl Transferase and deacetylase (HAT; HDAC) for the acetyl group addition/removal equilibrium (Li et al., 2014). Chromo-and bromo-domains are also present in chromatin remodelers, proteins that possess a nucleosome remodeling capability and act on higher order chromatin (Lauria and Rossi, 2011). In addition, in nucleosomes canonical histones can be replaced by histone variants with the aid of histone chaperones. Histone variants diverge from canonical histones for a different amino acid sequence and, being subjected to specific PTMs, are able to alter the chromatin structure, influencing the nucleosome dynamics and consequently the transcriptional regulation (Li and Fang, 2015). Finally, PTMs can also collaborate with DNA methylation processes establishing regulatory loops that allow specific chromatin states to be reinforced, as demonstrated for CHG and CHH methylation and H3K9me2 modification (see above; Du et al., 2012; Jiang and Berger, 2017).

In the last few years many studies focused attention on the role of histone PTMs on fruit development, considering a potential role in the crosstalk between chromatin modifications and *trans*-acting DNA-binding transcription factors (TFs) involved in fruit patterning. The expression profile of enzymes responsible for PTMs has been determined in many fruit species, suggesting their potential role in specific moments of growth, in both early and late fruit development (described below; Janssen et al., 2008; Aquea et al., 2010; Cigliano et al., 2013). Functional analyses that have contributed to attribute a function to histone modifiers in fruits were performed mainly in tomato model: for example the PRC2 Polycomb repressive complex is composed by different subunits with specific functions among which the deposition of H3K27me3 mark, with a fundamental role in fruits shape, flower formation, cutin accumulation, and fruit shelf life in tomato (How-Kit et al., 2010; Liu D. D. et al., 2012; Boureau et al., 2016; Liu et al., 2016). In Rosaceae family, the important role of PcG proteins, in relation to specific agronomic traits, was described: in particular, by the ectopic expression in tomato of a PRC2 PcG protein homolog *Malus hupehensis* FERTILIZATION-INDEPENDENT ENDOSPERM (MhFIE) leads to a co-suppression of the tomato homolog protein, resulting in various tomato phenotypes similar to those described in How-Kit et al. (2010), Boureau et al. (2016), and Liu et al. (2016).

## Rosaceae Fruit Growth Patterning

Because of the wide Rosaceae species phylogenetic diversity, translating into a large number of fruit types with different anatomical and physiological properties, three subfamilies are always referred to, dictated mainly by fruit structure: the Rosoideae (*Rosa, Fragaria*, and *Rubus* with hip, achene and berry fruit, respectively), Prunoideae (*Prunus* with drupe), and Maloideae (*Malus* and *Pyrus* with pome) (Potter et al., 2002). To simplify this complicated context and to translate the theoretical information obtained from genomic studies into agronomic practice, it is necessary to identify for each Rosaceae subgroup reference models. Although comparison of specific nuclear and chloroplast DNA loci allowed a new subfamily classification to be established (Potter et al., 2007), for the Rosaceae family it is currently possible to define three best-developed model species: the diploid strawberry (*Fragaria vesca*), peach (*Prunus persica*), and apple (*Malus* × *domestica*) (**Table 1**).

The standard botanical definition of fruit is complete maturity of the ovary with or without fused carpels (recent and ancestral fruit, respectively), and can be related only to Angiosperms. Fruit with fused carpels can also be classified as dry (indehiscent or non-dehiscent) or fleshy fruits, and derive only from carpels (‘true fruit’) or from accessory structures (‘false fruit’). In this articulated scenario, it is possible to collocate peach, apple, and strawberry, the three Rosaceae model plants that, on the basis of their specific fruit development pattern, result in the three different fruit types: drupe, pome, and achene, respectively (Seymour et al., 2008, 2013). Briefly, peach is the genetic and genomic reference for *Prunus* spp. harboring drupes characterized by a lignified endocarp (stone or pit), in which seeds are enveloped, and a fleshy and juicy mesocarp surrounded by an epicarp tissue. Apple, the most important deciduous tree fruit crop grown around the world, has a pome fruit structure, defined as ‘false fruit,’ in which the floral receptacle is the fleshy edible tissue (named cortex), and where the typical organization of pericarp (endocarp, mesocarp, and epicarp) characteristic of drupe is not present. Finally, the third typical Rosaceae family model is well represented by *Fragaria* spp. where the true fruit (achene) is localized on an accessory structure comprised of a fleshy receptacle (Seymour et al., 2013). It is important to underline that despite the diploid *F. vesca* is considered, for genetic and genomic reasons, the model species for *Fragaria* spp., actually the most largely cultivated species resulted the octoploid *Fragaria* × *ananassa* with (epi)genetic/genomic peculiar properties different from the model reference (Hirakawa et al., 2014) and discussed later in this review. Although the Rosaceae family offers a plethora of fruit development patterns, the main steps driving fruit set from fertilization to ripening are common to the different fruit models, involving a complex set of molecular and physiological events responsible for achievement of the final product.

**FIGURE 1:**
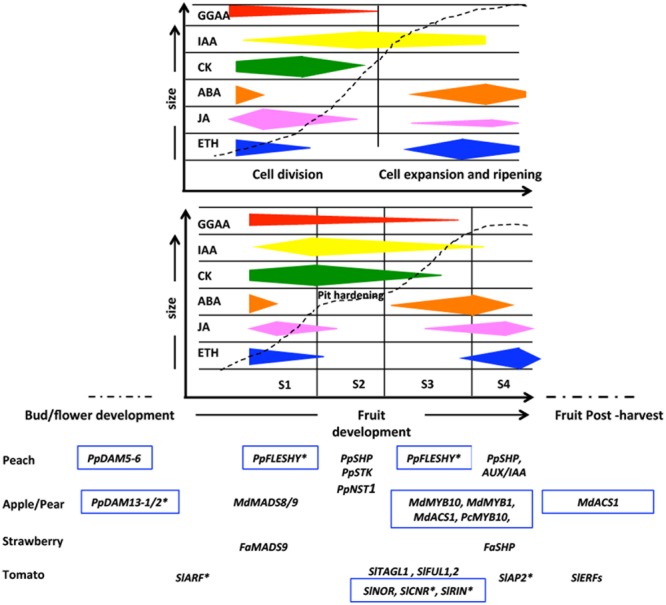
Schematic representation of main stages of fruit growth characterized by a sigmoid (top) and double sigmoid growth pattern (bottom) corresponding to pome and drupe fruit type, respectively. The fruit size and fruit developmental time are indicate in *y* and *x*-axis, respectively. The bud development and the post-harvest periods are also showed. The time points of involvement of plant hormones, TFs and epigenetic mechanisms influencing the different stages are showed in the chart. S1, S2, S3, and S4 indicate the four typical fruit growth phases in a double sigmoid model growth pattern. Hormonal content trends were drawn on the basis of data reported by Rasori et al. (2010) for drupe (e.g., peach fruit) and that from Kondo and Tomiyama (2000), Lara and Vendrell (2000), Eccher et al. (2008), and Devoghalaere et al. (2012) for pome (e.g., apple fruit). The genes with an epigenetic control are boxed and the genes with a post-transcriptional gene silencing mediated by microRNA are highlighted with an asterisk.

The fruit set and its development is concomitant with the embryo and seed formation and like the vast majority of flowering plants also Rosaceae plants share a life cycle alternating between a dominant diploid sporophytic and a short haploid gametophytic phase, defining the three main developmental stages characterized by different dynamics of plant sexual reproduction: sporogenesis, gametogenesis, and embryogenesis (Feng et al., 2013; She and Baroux, 2014). On the contrary, the following stage of fruit growth depends mainly on the fruit type, but focusing attention on fleshy fruits it is possible to describe the fruit development according to two main models, following either a single or a double sigmoid curve (**Figure 1**). The former is typical of pome fruits, whereas the latter of drupes. This simple representation has been improved by means of modeling approaches able to establish that for apple fruit (a pome) an expolinear equation, based on fruit diameter, is the one showing the best fit to its initial exponential growth phase followed by linear growth (Lakso et al., 1995). For peach fruit (a drupe), a mathematical analysis of fruit growth kinetics allowed it to be divided into four main developmental stages, named according to the parameter measured for calculations (cross diameter, fresh weight, and dry weight) as S1, S2, S3, and S4, FW1, FW2, FW3, and FW4, or DW1, DW2, DW3, and DW4, respectively, and the duration of the different developmental stages varies according to the genotype (Chalmers and Ende, 1975).

Concerning the early stage of development (S1), the length of cell division plays a relevant role in determining the final fruit density (number of cells per volume) at harvest, as its storability and nutritional value are strongly linked to this parameter. In turn, cell division depends upon the final size of the fruit. Therefore, this stage is very short or absent in small fruits (i.e., in raspberry), while it lasts for 7–25 and 21–35 days in peach and apple, depending on the variety (Fishman and Génard, 1998). It is well known that phytohormones such as auxins, cytokinins (CK) and gibberellins (GGAA) control the cell cycle, in particular through the CDK/cyclins involved in G1/S and G2/M transitions (McAtee et al., 2013). In addition to the cell division process, cell expansion, occurring at late S1 and S3, is linked to an increase of both vacuole and cell wall volume and also deeply influenced by phytohormones promoting cell growth such as GGAAs and auxins (Kumar et al., 2014). The increase of cell volume within the fruit pericarp is clearly correlated to the mean ploidy level of various Rosaceae fruits, and a positive correlation between endoreduplication level and size of final fruit was found (Chevalier et al., 2011). In drupes, S2 is characterized by pit hardening, starting from the apical side of the fruit and progressively expanding until reaching the opposite side. Endocarp cells can already be recognized at S1, because of the phenol compounds accumulating before lignin formation (Masia et al., 1992). Lastly, S4 is the stage where fruits acquire the prerequisite competence to enter the final developmental stage, i.e., ripening (Bonghi et al., 2011). During ripening important structural and biochemical changes occur that result in the conversion of a less palatable green fruit into a highly palatable and nutritionally rich one. Fleshy fruits are classified physiologically as climacteric and non-climacteric and Rosaceae fruits mainly belong to the former category. Climacteric fruits are apple, apricot, plum, and peach and they show a concomitant increase in respiration and ethylene biosynthesis during ripening. However some plum cvs show non-climacteric behavior (also called suppressed climacteric) where these two events are lacking. In both categories the progression of ripening cannot be stalled and generally leads to over-ripening characterized by physiological events that can negatively affect fruit structure and composition and consequently leads to products being discarded. For this reason the reduction of post-harvest losses is the main goal for agronomic research focused in the control of fruit chain management.

As previously reported, plant hormones play a key role during the whole fruit development and ripening processes (Klee and Giovannoni, 2011; Seymour et al., 2013; Kumar et al., 2014), and an emerging elucidation in understanding the possible relationship between them and epigenetic modifications and how they influence toward each other, is increasing. In particular several current evidences support the fact that hormones coordinate fruit growth and ripening throughout a complex transcriptional network, in which hormonal-related transcripts such as specific class of TFs belonging to Auxin Responsive Factors (ARFs), basic helix-loop-helix (bHLH), Ethylene Responsive Factors (ERFs), MADS-box, NAC, and WRKY families (Seymour et al., 2013; Karlova et al., 2014) and transcriptional repressors of hormone-regulated genes (e.g., AUX/IAA or DELLA proteins, Azzi et al., 2015) play main roles. Recent studies highlighted that the transcription of TFs and transcriptional repressors is controlled by microRNAs (miRNAs) and deposition of specific epigenetic marks related to chromatin remodeling (see below and Yamamuro et al., 2016) (**Figure 1**). In this framework, we have surveyed the literature published on the transcriptional modulation of fruit development regulators, focusing our attention on TFs whose mRNA levels can be modulated at post-transcriptional level by miRNAs and by possible epigenetic mechanisms. In recent years, many miRNAs have been claimed to control fruit growth and development by controlling the interplay between hormones at signaling pathway level (Curaba et al., 2014) as well as the accumulation of mRNAs coding for TFs (Solofoharivelo et al., 2014; Ripoll et al., 2015). A further level of complexity is due to the epigenetic control on the miRNA promoter (Meng et al., 2011), and, although this control is particularly active for miRNAs involved in the response to environment conditions (Yong-Villalobos et al., 2015) there is some evidence suggesting the existence of a similar mechanism for reproductive events such flower and fruit development (Song et al., 2015).

## Fruit Development and Epigenetic Control: A Crosstalk Step By Step

For many years the breeding programs have been based on a systematic marker-assisted introgression of quality traits, and for some varieties of several crops by simple phenotypic selections (Collard and Mackill, 2008), however, these approaches make difficult to explain the high level of phenotypic variability (Ball, 2013). An epigenetic approach could be therefore the key to understanding the relationship between changes in the environment and correlated variations in gene expression resulting in phenotypic variability observed in many plant developmental processes (Róis et al., 2013; Xu et al., 2013). In this context, the identification of factors involved in epigenetic mechanisms and acting as regulatory checkpoints in environment perception and gene response is a start point for a better understanding of a complex process as fruit development and ripening.

Taking into account these notions, in this review we explore the epigenetic mechanisms influencing key developmental stages of Rosaceae fruit crop models to compare these different systems with each other and also in relation to data reported for tomato. In particular, a first long initial phase from fruit set up to reaching final size and a second late phase including the ripening and post-harvest processes is discussed.

### The First Phase: From Floral Bud to Ripe Fruit

Development of fleshy fruits involves a profound phase change in the leaf-like tissues that are going to encase or be associated with the mature seeds, characterized by a wide-ranging alteration of the metabolic state of carpel organs and associated tissues. In the transition/development of Rosaceae flowers into fruits, similarly to other botanical families, a complex set of molecular circuits occurs mainly mediated by key players belonging to the MADS-box family of TFs. Among these, in many perennial Rosaceae species the *Dormancy-associated MADS-box* genes (*DAMs*) have been identified as the internal factors controlling the development starting from bud endo-dormancy regulation (Li et al., 2009; Horvath et al., 2010; Sasaki et al., 2011), and their epigenetic control was also determined in some Rosaceae fruit such as apricot (Sasaki et al., 2011), peach (Li et al., 2009; Leida et al., 2010, 2012), apple (Mimida et al., 2015), and pear (Liu G. et al., 2012). In Japanese pear and peach for example, some members of this gene family have the same transcriptional trends during this temporary development window: an up-regulation of both genes *PpDAM13-1/2* and *PpDAM5/6*, in pear and peach, respectively, toward endo-dormancy establishment was observed, followed by their down-regulation during endo-dormancy release (Li et al., 2009; Ubi et al., 2010; Yamane et al., 2011; Saito et al., 2013) (**Figure 1**). Regarding the role of histone modifications, in some peach cultivars an accumulation of H3K27me3 epigenetic mark associated with a repression of the gene transcription was suggested, together with decreased levels of H3K4me3 histone modification, leading to an endo-dormancy establishment/maintenance/release equilibrium (Leida et al., 2012). Similar results were observed also in *Pyrus pyrifolia*, where a decrease of H3K4me3 accumulation translating with the down-regulation of *PpDAMS13-1* was related to endo-dormancy release (Saito et al., 2015). In addition, it was demonstrated that DNA methylation changes associated to low temperatures have a role in dormancy period and its release in many other crops. In Royal delicious apple varieties 47 methylation sensitive DNA fragments associated to genes, involved in diverse physiological and regulatory pathways, were identified in various developmental stages during dormancy and fruit set, confirming the possible involvement of this epigenetic mechanism also in regulation of catalytic/cellular/metabolic processes in response to environmental stimuli (Kumar et al., 2016).

Recent discoveries indicate that a strict genetic and epigenetic control influences the entire fruit development process starting from seed formation. Indeed several pieces of evidence suggest a dynamic reprogramming of the chromatin states already in first stages of the fertilization process, with a global remodeling of the meiocyte chromatin state, by the establishment of an equilibrium between permissive-associated marks like H3K4me3 and H3K9ac and the repressive-related marks including H3K27me1, H3K27me3, and H3K9me1 (She et al., 2013; She and Baroux, 2014). This is what happens during seed formation, but what happens around the seed? Studies performed in model *A. thaliana* have allowed to define the pivotal roles of specific MADS-box genes during fruit development. In detail genes such as *AGAMOUS* (AG) and *SEPALLATA* (SEP) are responsible of carpel fusion during the first steps of fruit set, confirming their fundamental attendance in floral structures definition (Scutt et al., 2006). Their expression appears conserved throughout the Angiosperm lineage, including monocots, and seems to be functionally redundant with that of other TFs MADS-box members, like *APETALA1* (AP1) and *FRUITFULL* (FUL): their functions overlap in floral meristems specification but diverge in later developmental stages during fruit growth (Ferrandiz et al., 2000; Scutt et al., 2006). In fact, many of them in addition to having a role in carpel development, participate in different phases of fruit patterning and their importance is testified in tomato (*SlFUL1, 2; SlTAGL1; RIPENING INHIBITOR, SlMADS-RIN*) apple (*MdMADS8/9*), strawberry (*FaMADS9, FaSHP*), peach (*PpSHP*) as reported by Karlova et al. (2014), from the earlier developmental phase up to after fruit growth.

However, genetic studies performed in Arabidopsis have highlighted that TFs belonging not only to the MADS-box class but also to bHLH family are actively involved in the regulation of fruit development (Liljegren et al., 2000, 2004). Moreover the isolation in tomato of orthologs TFs genes controlling the silique formation and maturation in Arabidopsis, confirmed that their targets are mainly promoters of genes necessary for fleshy fruit development/ripening. This high correlation among different TFs involved in same pathways but in completely different fruit systems was confirmed in peach, where a high analogy, in terms of TFs involved (e.g., *SEEDSTICK, STK, SHATTERPROOF, SHP, SECONDARY WALL THICKENING PROMOTING FACTOR, NTS1*), between the regulation of the lignification in silique valve margin in Arabidopsis and the hardening of endocarp in peach was observed (Dardick et al., 2010; Dardick and Callahan, 2014). In particular the characterization of peach *HEC3*-like gene *FLESHY*, showing a double function in channeling the phenylpropanoids pathway to either lignin or flavor/aroma, together with its possible role in triggering auxin-ethylene cross talk at the start of ripening, suggested the hypothesis, also in drupe patterning determination, of epigenetic control defined by chromatin specific epigenetic marks deposition (Botton et al., 2016).

In tomato fleshy fruit model system, elucidation of the genetic basis regulating the early and late steps of fruit growth has confirmed how the establishment of peculiar gene regulation patterns and genome stability could be epigenetically controlled. Genes encoding histone-modifying enzymes, in particular *HMT*, are expressed preferentially during early fruit developmental phases, confirming the involvement of histone methylation during cell division phase. Similarly, genes encoding HAT and HDAC are specifically expressed during early apple and grape fruits developmental stages, confirming their preferential expression in precise fruit growth stages (Janssen et al., 2008; Aquea et al., 2010, 2011; Cigliano et al., 2013). Moreover, a general DNA demethylation was observed during fruit growth together with the activation of specific pathways correlated with the beginning of fruit ripening, influencing the genome stability and consequently gene expression (Zhong et al., 2013; Liu et al., 2015). Interestingly, this DNA demethylation occurs in a temporal window characterized by a limited endoreduplication process, typical in fleshy pericarp tissues, reducing the probability of its passive loss depending by DNA replication (Teyssier et al., 2008). In apple for example a negative correlation between methylation and anthocyanin content at skin level was observed in an anthocyanin-deficient yellow-skin somatic mutant. This mutant showed altered methylation levels in two promoter regions of the *MdMYB10* TF. In detail, a high level of DNA methylation alongside fruit development in the mutant was observed, in opposition to a low and constant level in a wild-type cv, where it negatively correlated with both anthocyanin content and expression of *MdMYB10* itself (El-Sharkawy et al., 2015). The study of specific epigenetic modifiers in other crop is crucial for understanding the influence of these epigenetic regulatory mechanisms not only in climacteric but also in non-climacteric fruit systems. In *F. vesca* a possible global variation in both DNA methylation and histone methylation was suggested: during the fruit ripening, and in particular in the pink stage transition, a severe changing of DNA methylation was testified together with a peak of expression levels for genes such as histone methyltransferase and demethylase (Gu et al., 2016).

### The Second Phase: From Fruit Ripening to Post-harvest

The market value of many plants products depends often by several factors. In *primis* the choice of specific cultivars for each species may influence the success of final product, such as the fruit, to thanks their high quality traits. But this is not always sufficient, in fact, several cultural practices, including the harvest, are linked to the crop used and may contribute or not to an added commercial value (Srancikova et al., 2013). For this reason in the fruit development cycle, the period including final ripening, harvest and post-harvest phases is gaining increasing importance in crop research and molecular studies, because various stresses that affect the fresh product can be the cause of reduced quality and health of the product (Ansari and Tuteja, 2013). To cope and overcome these physiological problems, specific managements associated to an implemented knowledge, are needed to avoid enormous quality loss. Results in the literature have helped in the understanding of the physiological and molecular aspects particularly of ethylene-related stresses, which influence fruit quality at post-harvest (Ansari and Tuteja, 2013, 2015; Singh et al., 2013). Several studies report molecular approaches to define the epigenetic influence during these later stages (ripening and post-harvest) confirming its importance in controlling both physiological and biochemical events, despite the different fruit anatomical structures characterizing several model systems of the Rosaceae family (**Figure 1**). To date, also in this case, much of the information on the genetic regulation and epigenetic control of TFs involved in later fruit growth stages are mainly elucidated in tomato. Attention is mainly focused on MADS-RIN, *NON*-*RIPENING* (NOR; also called NAC-NOR), and *COLORLESS NON*-*RIPENING* (CNR; also called SPL-CNR) considered as key TF genes for the regulation of fruit growth/ripening transition phase (Vrebalov et al., 2002; Manning et al., 2006; Giovannoni, 2007). A large-scale identification of direct RIN targets by chromatin immunoprecipitation coupled with DNA microarray analysis (ChIP-chip) allowed the predicted target promoters of these tomato proteins to be identified (Fujisawa et al., 2013). Epigenetic regulation of these main actor genes was also supported and verified: a reprogramming of gene expression by active DNA demethylation was assumed to exert a greater role in controlling ripening initiation. In support of this, recent works describing the methylome dynamics in tomato fruit pericarp revealed substantial changes in the distribution of DNA methylation over the tomato genome during fruit development, and in particular at specific promoters, such as the NOR and CNR promoters during ripening (Manning et al., 2006; Zhong et al., 2013). This general demethylation, mediated by a tomato DEMETER-like DNA demethylase (SlDML2) supports the idea that active DNA demethylation governs the switch on of ripening in tomato pericarp (Liu et al., 2015). Taking the role of DNA methylation in Rosaceae into account, some evidences suggest that there is a direct involvement in the regulation of fruit ripening in this family (Wang et al., 2013). The silencing of pear MYB gene (*PcMYB10*), caused by a hypermethylation at promoter level, is correlated with the formation of green-skinned spot, suggesting a putative methylation-dependent mechanism in the regulation of anthocyanin biosynthesis during pear ripening (Wang et al., 2013). Moreover, in Honey Crisp apple fruit, a similar hypermethylation in *MdMYB10* gene promoter is responsible of a striped pigmentation (Telias et al., 2011). On the contrary a decrease on DNA methylation in 5′ upstream region of two non-transcribed alleles of MdMYB1, an R2R3-Type MYB gene involved in apple anthocyanin synthesis, was also observed. In detail, a switch on of transcription, mediated by a low level of DNA methylation and H3K27me3 mark together to an higher levels of H3ac and H3K4me3 histone marks, was measured in the skin of the fruit immediately after bag removal, a commonly used technique for enhancing the red pigmentation of fruit skin (Bai et al., 2016). Also in *F. vesca* it was observed that transcriptional levels of genes, such as DNA methylase and demethylase, achieve their highest values during the fruit color change from white to red, supporting and confirming further the idea of a fundamental involvement of DNA methylation in specific biological processes like fruit ripening (Gu et al., 2016). These experimental evidences indicate that the balancing between DNA methylation and active DNA demethylation is a novel key component of the ripening switch that can lead to the transactivation both of genes positively correlated to ripening, as the ripening TFs, and rate-limiting enzymes driving biochemical processes (Liu et al., 2015). To support this view, Zhong et al. (2013) demonstrate that immature tomatoes treated with a methyl transferase inhibitor were able to start the ripening program. Moreover, the expression analysis of genes involved in methylation pattern maintenance indicated that *MET1, CMTs*, and many *SlDRMs* are mostly involved during early fruit development while *SlDRM7* expression peaks during early phases of fruit ripening (Teyssier et al., 2008). In a articulated scenario like a fruit, this highly methylated state could result necessary to prevent a premature fruit ripening before seed formation, gaining the function of timing synchronizer (Gallusci et al., 2016).

In post-harvest research almost all epigenetic studies are focused on the modulation of pathways involved in senescence (Osorio et al., 2013; Xu et al., 2013; Cherian et al., 2014) and abiotic/biotic stress responses (Jones-Rhoades and Bartel, 2004; Dowen et al., 2012). DNA methylation correlated with chromatin structure modifications are involved in controlling these post-harvest physiology events. Studies on tomato provided insight into processes associated with post-harvest traits related to cuticle characteristics, since the fruit surface properties, associated to cuticle biosynthesis and its deposition, are important traits defining the fruit quality in an economical context. For this reason the cuticle biosynthetic pathway has been well defined in last decades, even if it was considered mainly a canonical influence by phytohormones and the regulatory mechanisms are only now beginning to be elucidated at (epi)genetic level. The involvement of MADS-box TFs belonging to the APETALA2 homeodomain-leucine zipper IV and MYB families represent important examples of regulator genes involved in cuticle biosynthesis and epidermal cell differentiation, supporting the idea that have a possible epigenetic control (Hen-Avivi et al., 2014). In addition to these molecular observations regarding cuticle deposition in tomato, several other observations confirmed an interplay between hormones and possible epigenetic mechanisms in this final developmental event. In particular, an epigenetic influence on MADS-RIN and its regulon, including genes involved in ethylene biosynthesis and action, was well documented and described in tomato system by Liu et al. (2016). Also in Rosaceae family a DNA methylation analysis at ACS1 promoter level in apple fruits confirmed that ethylene biosynthesis, that usually occurs during post-harvest and is linked to abiotic stresses, could have an epigenetic control in relation to the internal ethylene concentrations (Gapper et al., 2013). However, despite the ethylene role is by far the most investigated during the entire developmental cycle with a pivotal role in both fruit growth and ripening of fleshy fruit, including the Rosaceae fruits, the involvement of other plant hormones and their putative epigenetic influence has well been described. Plant hormones such as auxin, GA, and brassinosteroids may modulate epigenome influencing modification as de/acetylation or de/methylation of histones and on the contrary these modification have also been implicate in the regulation of hormone responses in plants (Yamamuro et al., 2016). The role of chromatin structure in gene regulation during post-harvest is better testified in species that do not belong to Rosaceae family, by examples of chromatin remodeling-related proteins identified and involved in stress responses associated to the post-harvest process, including high susceptibility to biotic and abiotic stresses. Their role is generally performed through the interaction between chromatin-related proteins, such as histone modification enzymes, components of chromatin remodeling complex and linker histone H1 and relative TFs (Kim et al., 2010; Zhu et al., 2011). This is the case of histone deacetylase HD2 characterized in longan fruit. HD2 is able to mediate transcriptional repression in many biological processes and in longan fruit it was shown to physically protein interact with the ethylene-responsive factor-like gene (*ERF1*) that is involved in fruit post-harvest related senescence (Kuang et al., 2012). Similarly, by yeast two-hybrid and Bimolecular Fluorescence Complementation assays in banana, a direct interaction between linker histone H1 gene (*MaHIS1*) and a WRKY TF was detected in response to ripening and stress responses, improving also the limited information on linker histone H1 in fruits (Wang et al., 2012). A general involvement of histone modification genes (*HMs*) in sweet orange during the whole pre-harvest fruit development and post-harvest was reported following the genome sequencing of this species. In particular a genomic analysis highlighted chromosome locations, phylogenetic comparison and gene structures of HM members during biotic stress induced by blue mold infection responses (Xu et al., 2015). Finally, in pear the Cold Temperature Conditioning (CTC) process, applied often in post-harvest to develop ripening capacity, in turn triggered the onset of the ripening process and the switch off of some positive transcriptional regulators, namely SW/SF genes associated to ripening itself. Their involvement both in chromatin remodeling and acquirement of developmental competence, suggests and confirms once again the key role of chromatin structure in ripening initiation and fruit quality preservation in post-harvest as well (Reyes, 2014; Jajo et al., 2015).

## Final Remarks and Future Perspectives

The results presented and discussed here indicate that to obtain better knowledge on the intricate process of fruit development, at both physiological and molecular level, it will be important to succeed in translating the information acquired up to date in model systems (e.g., tomato) to other crops. As reported above, the Rosaceae family comprises several species with a high agronomic value but with limited knowledge about the main molecular actors involved in fruit development.

To fill this gap following aspects should be considered:

(1) Although many molecular mechanisms underlying fruit development have been investigated and their genetic and, in some cases, epigenetic regulation determined, a complete view of the process needs the application of methodologies supplied in ‘omics’ era also to Rosaceae crops. Different genome-wide approaches may be considered as potential suppliers of epigenetic information regarding fruit development, with the aim of completing the genetic puzzle until now available only for some species. DNA methylome and chromatin states analyses (by BSEQ and ChIP-SEQ, respectively) could be two fundamental goals enrich and improve the list of key genes, already available (see **Table 2**), with an epigenetic role or regulation in fruit development, ripening and post-harvest.
(2) DNA methylation and histone mark studies in different plant tissues and the comparison of their patterns with transcriptomes of different cell types will be pivotal to determine whether epigenetic states directly influence gene expression at specific loci involved in fruit developmental processes.
(3) The knowledge of the logic and temporal sequence of the chromatin state changes that are responsible of the repression and/or activation of new gene expression programs appear essential to better understand developmental transition in fruit development in the context of chromatin.

**Table 2:** Schematic representation of Rosaceae model species with a putative epigenetic control on specific target genes during biological processes related to fruit development.

Species	Physiological process	Epigenetic modification	Examples of target genes
Peach	Endo-dormancy release Fruit patterning	Histone modification	*DAM5*/6 *Fleshy*
Apple	Anthocyanin synthesis Ethylene synthesis	DNA methylation	*MYB10 ACS1*
Strawberry	Fruit ripening	DNA methylation Histone modification	**_**

*Since a long time the three different fruit systems are considered like developmental models in Rosaceae family for their peculiar fruit patterning and growth, but recently they have been also taken into account for epigenetic studies related to specific growth and physiological stages. In peach and apple, specific target genes with an epigenetic control and involved in these developmental processes are also defined and described.*

These focus points should be faced taking into account that the majority of Rosaceae spp. are vegetatively propagated and have multiple complete sets of genetic information (polyploidy). It is well known that the majority of Rosaceae species are vegetatively propagated, bypassing the meiotic process and seed formation. Since many of the genes that control important quality traits of agronomic interest could be epigenetically regulated, it will be necessary to evaluate if the specific epigenetic marks influencing their regulation, are or are not maintained following the typical and canonical agronomic practices of plant propagation, such as clonal propagation (for cutting) or grafting. In both cases, the maintenance of peculiar epigenetic marks in the form of epialleles, controlling specific fruit quality traits, is an emerging aspect to consider in arboriculture research, together to the understanding of which potential epialleles are maintained during plant propagation and how could provide a new view in molecular selection strategies. In grafting practice, which results the most common mean to clonally propagate desirable Rosaceae tree fruit cultivars (e.g., for apples, pears, and peaches) the elucidation of ‘molecular’ interaction between scion and rootstock is a new frontier to cross, to determine whether this agronomic tool is able to transmit heritable (epi)genetic changes in the scion (and *vice versa*). It is well known that RNA gene silencing may be transmissible in up and downward direction determining modifications in DNA methylation levels in receiving tissues (Molnar et al., 2010; Lewsey et al., 2016). In Solanaceae family, for example, it has also been shown that an interspecific grafting led to heritable changes in DNA methylation both in scion and rootstock, suggesting a reciprocal exchange of molecular information (Wu et al., 2013). Furthermore, multiple research studies support that a long-distance transport of precursor and mature sRNAs, working as signals molecules, is able to elicit numerous physiological effects in both bionts (Pant et al., 2008; Molnar et al., 2010; Jensen et al., 2012; Bhogale et al., 2014).

In relation to the important role of sRNAs, in particular miRNAs many interesting aspects concerning their involvement in plant development have emerged from many molecular research works. As mentioned above, it is well proven that many genes involved in fruit set/development/ripening processes are regulated through post-transcriptional gene silencing (PTGS) mechanisms mediated by miRNAs: the first study designed to investigate miRNAs in fleshy fruits was done on tomato, where 9 conserved and 12 novel miRNAs expressed in fruit were identified (Pilcher et al., 2007). This number was soon increased to include miR168 (inhibiting *ARGONAUTE1*), miR172 (inhibiting *APETALA2*) (Itaya et al., 2008), and miR156 (targeting *CNR*) (Zhang et al., 2011). In addition, several members of *ARF* family predicted to be targets of miR160, and in particular miR167 (whose target is the *ARF8*) (Mallory et al., 2005). In turn, it was also observed that many TFs, known as regulators of developmental processes, directly control the transcription of *MIRNA* genes: for example, in tomato the direct binding of MADS-RIN to the promoter of MIR172a gene was demonstrated, showing a close correlation between miRNA/RIN transcript level and ethylene pathway, because the regulatory roles of miRNAs and MADS-RIN TF during fruit development and ripening in relation to ethylene response were elucidated (Gao et al., 2015). In *P. pyrifolia*, miRNAs are involved in the control of *DAM* genes expression. In particular, by degradome data analysis, *PpDAMs* transcript targeting and degradation by miR6390 was shown to allow the release of *PpFT2* that is responsible of endo-dormancy regulation (Niu et al., 2016). Interestingly, recent data have indicated a possible epigenetic regulation of MIRNA transcription through histone PTMs. In Arabidopsis the deposition of H3K27me3 mark directly represses only specific TF families, indirectly activating other TFs through H3K27me3-mediated silencing of MIRNA genes. For these reasons H3K27me3 represents one of the main epigenetic marks able to determine specific expression patterns tissue-dependent and targeting genes involved in auxin response (perception, biosynthesis and signal transduction) (Lafos et al., 2011). In our recent research we have demonstrated that also in peach fruit, a putative post-transcriptional regulation of the TF *FLESHY* is likely to be mediated by miR710, confirming the relevant involvement of MIRNA loci in controlling the correct development of fruit (Botton et al., 2016). To confirm the possible role of PTMs in controlling of miRNA genesis the chromatin state analysis on MIRNA locus is under investigation.

Lastly, an additional point to consider is the presence of numerous polyploid species in Rosaceae family, even within the same genus, as well testified by *F. vesca* (diploid) vs. *Fragaria* × *ananassa* (octoploid). Often the polyploid condition is associated to a mosaic expression profile, which involves homologous genes of different genomes. This expression variability can be driven by epigenetic gene silencing mechanisms, as reported for genus Rosa (Khaitová et al., 2010) and can contribute to create an high phenotypic variability which is potentially useful to breed new varieties. For these reasons it is explainable the high economic and agronomic interest toward the polyploid species (e.g., *Fragaria × ananassa*), despite sometimes for them the interpretation of epigenetic regulatory mechanisms results difficult.

In conclusion, deepening the knowledge of the genetic and epigenetic processes regulating Rosaceae fruit development, with the aid of new ‘omics’ tools and developing innovative research approaches could represent an important priority for fruit crop improvement.

## Author Contributions

SF, SV, and CB conceived and wrote the manuscript. AR helped in figures. All authors revised the MS and approved it for publication.

## Conflict of Interest Statement

The authors declare that the research was conducted in the absence of any commercial or financial relationships that could be construed as a potential conflict of interest.
